# Social influences on the occupational dietary behaviour of firefighters: A scoping review

**DOI:** 10.1111/1747-0080.70076

**Published:** 2026-04-20

**Authors:** Holly Ranson, Megan Lee, Robin Orr, Louise Van Herwerden, Kristen Mackenzie‐Shalders, Amy Bannatyne

**Affiliations:** ^1^ Faculty of Health Sciences & Medicine Bond University Gold Coast Australia; ^2^ Faculty of Society and Design Bond University Gold Coast Australia; ^3^ Tactical Research Unit Bond University Gold Coast Australia

**Keywords:** eating behavior, first responders, social norms, systematic review, workplace environment

## Abstract

**Aim:**

This scoping review aims to explore the literature on social influences on firefighters' occupational dietary behaviour.

**Methods:**

Academic databases (PsycINFO, Web of Science Core Collection, Scopus, PubMed, Embase, ProQuest Dissertations & Theses) were searched from inception to 29 August 2025, for published articles and theses meeting the inclusion criteria: English language, primary research studies reporting on social influences on workplace dietary behaviour in international firefighters aged 18–65 years.

**Results:**

Of 2576 identified works, 24 met the eligibility criteria; 20 peer‐reviewed studies and four PhD dissertations. Ten qualitative, nine quantitative, and five mixed‐methods studies were included. Twenty‐one studies were conducted in North America, with the remainder conducted in Australia, Canada, and the United Kingdom. Sample populations included career, volunteer, and wildlands firefighters (*n* = 14–599), who were predominantly Caucasian males with a median age between 39 and 51 years. The volume of evidence suggests the social influence of colleagues significantly shapes firefighters' dietary behaviour. This influence can be negative (promoting less healthy eating patterns) or positive (promoting healthier eating patterns). Firefighters who exert social influence and those most susceptible to influence tend to exhibit different characteristics. Social influence also plays a key role in communicating normative behaviour, contributing to a distinct culture in firefighting workplaces.

**Conclusions:**

The social influences on firefighters' occupational dietary behaviour warrant further investigation as a potential barrier or facilitator to dietary change. Given diet is a modifiable risk factor for health conditions disproportionately affecting firefighters, understanding these influences is critical to informing effective interventions.

## 1 INTRODUCTION

Nutrition is a modifiable risk factor for the development of chronic disease and health conditions such as obesity, cardiovascular disease, depression, and some cancers.^1^, ^2^, ^3^ Firefighters experience these conditions at a higher rate than the general population.^3^, ^4^, ^5^, ^6^ At work, firefighters are known to consume diets that are high in saturated fat, salt, sugar, and red meat and low in fruit, vegetables, nuts, fish, whole grains, and fibre.^7^, ^8^, ^9^ They are reported to self‐serve oversized portions, eat quickly, and snack on energy‐dense foods and caffeinated beverages throughout their shifts.^7^ Research suggests that firefighters' diets are poorer at work than at home,^10^, ^11^, ^12^ indicating that some aspect of the workplace may be influencing their dietary behaviour.

The term ‘dietary behaviour’ comprises three categories: food choice, eating behaviour, and dietary intake/nutrition.^13^ ‘Food choice’ represents terms such as preferences, product purchase and intentions; ‘eating behaviour’ encompasses eating habits, eating occasions, portions, dieting, fussy eating and disordered eating; and ‘dietary intake/nutrition’ refers to dietary and meal patterns, food intake, and the components of food including nutrients and energy.^13^

Peer pressure, the attitudes and decisions of colleagues, and fire station eating culture have been identified as occupational factors that can impact firefighters' dietary behaviour.^7^, ^14^ Firefighters work, sleep, eat, train, and spend down‐time together in their workplaces, often viewing one another more as ‘family’ than colleagues.^15^ This dynamic, coupled with the routine sharing of communal ‘family style’ meals, makes it unsurprising that they influence each other's dietary behaviour.^16^ We therefore hypothesise that the social influence of colleagues plays a significant role in shaping firefighters' dietary behaviour. Social influence is defined by the American Psychological Association (APA) as “any change in an individual's thoughts, feelings, or behaviours caused by other people, who may be actually present or whose presence is imagined, expected, or only implied”.^17^ Social influences have been included in models of food choice theory since the 1990s^18^ and remain an important determinant of individual food choices today.^19^ Hence, they have been identified as a key area for future research by the Determinants of Nutrition and Eating (DONE) framework.^20^ In the context of food and eating, social influences include phenomena such as social modelling, peer influence, groupthink, social norms, social facilitation, social identity and impression management.^21^, ^22^ The mere presence of others while eating has been shown to influence what and how much is eaten.^21^ Social influences have been found to shape the dietary behaviours of colleagues,^23^ including in other shift‐working^24^, ^25^, ^26^ and first responder^27^, ^28^ populations. Across these occupational groups, social influences operate as both enablers and barriers to healthy eating. Shared meals and snacks are used to build camaraderie, reduce stress, and cope with the monotony of night shifts, but often normalise high‐energy, convenience‐based food choices.^25^, ^29^ Conversely, role modelling, colleague encouragement and healthy peer norms can enhance fruit and vegetable consumption and decrease unhealthy eating.^23^, ^30^ Nurses, for example, report that conversations about diet and exercise with their colleagues can motivate healthier behaviours, while peer pressure to partake in the sharing of cakes, biscuits, and takeaway foods reinforces unhealthy norms.^26^, ^31^ In police and paramedic populations, the social influence of colleagues has been shown to drive unhealthy food choices during shifts, often overriding individual dietary intentions.^27^, ^28^, ^32^ In shift workers with type 2 diabetes, the desire to fit in and maintain social bonds through food sharing outweighs personal health goals.^24^ Overall, the literature highlights that in shift‐working and first responder settings, dietary behaviours are not merely individual choices but are strongly shaped by workplace culture, peer norms, and the social functions of eating.

These findings provide an important foundation for understanding the occupational dietary behaviours of firefighters, a group for whom communal eating is embedded in workplace culture, potentially amplifying the influence of social dynamics on dietary behaviour. Given the potential impact of dietary behaviours on a range of health outcomes, there is a clear need to explore this topic further. Due to the absence of prior systematic syntheses, a scoping review approach was chosen to provide an overview of the literature and identify key themes related to social influences on firefighters' dietary behaviours. This review seeks to:

- Explore the nature of social influence on dietary behaviour among firefighters.
- Describe the characteristics of individuals and positions of power who exert influence over dietary choices.
- Identify and describe the dietary behaviour social norms present in firefighting settings.

The anticipated output is a thematic overview that can inform the development of targeted interventions to enhance firefighters' occupational nutrition. The findings may be of value to other shift‐working professions such as paramedics, law enforcement officers, correctional staff, and nurses, who face similar challenges regarding dietary behaviour and health.^28^, ^33^

## 2 METHODS

A preliminary search of PubMed was conducted and no current systematic reviews or scoping reviews on the topic were identified. One narrative review had been conducted on peer‐reviewed literature on firefighters' dietary behaviour.^34^ This review, published in 2022, focused on a small subsection of the firefighter population (structural firefighters) and had a wide scope that included all factors affecting structural firefighters' dietary behaviour, as well as dietary intervention studies.

Based on the dearth of targeted research identified in the preliminary search, a scoping review was considered the most appropriate methodology to broadly and exploratively describe the nature of existing literature, rather than to address a narrowly defined research question. As the existing literature was not sufficient to make evidence‐based conclusions, a scoping review could identify knowledge gaps and emerging themes to guide future research. The scoping review used the Joanna Briggs Institute's (JBI) Population, Concept, and Context (PCC) methodology^35^ and the Preferred Reporting Items for Systematic Reviews and Meta‐Analyses extension for Scoping Reviews (PRISMA‐ScR) checklist^36^ to guide the reporting of the review. The scoping review protocol was registered with Open Science Framework (OSF) on 23 June 2023.^37^ The data were managed and stored using Covidence,^38^ an electronic systematic review platform.

A preliminary search of PubMed using the following terms ‘firefighter*’, ‘fire fighter*’, ‘food’, ‘diet*’, ‘nutrition’ and ‘eating’ was undertaken followed by analysis of titles, abstracts, and keywords. This information was used to build the search string with the assistance of the Faculty Librarian. The search strategy, including all identified keywords and index terms, was adapted for each included database using an online Polyglot search translator.^39^ One reviewer carried out a systematic search of six electronic databases (PsycINFO, Web of Science Core Collection [all editions], Scopus, PubMed, Embase, and ProQuest Dissertations & Theses) from inception to 29 August 2025. See Table S1 for the complete search strategy.

Identified works were imported into Covidence where duplicates were removed. Two reviewers independently screened the titles and abstracts of all studies identified through the search strategy, followed by full‐text reviews of potentially relevant articles, applying the pre‐defined eligibility criteria. Disagreements were resolved by consensus or by consulting with a third reviewer. Studies were included if they were: (i) peer‐reviewed primary studies or theses published in English; (ii) studies referring to social influence (as defined by the APA) on dietary behaviour; and (iii) the population was paid and volunteer international firefighters of any role, rank or length of service aged between 18 and 65 years. Articles excluded were removed and their reasons recorded. To identify additional articles, the reference lists of the remaining studies were reviewed. Any additional article identified was subjected to the same screening process as the initial articles.

Two reviewers independently extracted data from included studies using a data extraction table (see Table S1). Information extracted included: study author and year; aims of the research; study setting (e.g., geographical location of sample and type of fire station); sample size and characteristics (e.g., gender, median age, type of firefighter [career or volunteer] and length of service); study methodology; outcome measures and key findings. Qualitative synthesis was used to identify recurring themes, patterns, and insights occurring in the extracted data, with the results presented in narrative summary. One reviewer conducted initial, line‐by‐line coding, highlighting small segments of text that conveyed similar meanings. These initial codes captured concepts such as positive and negative influences, peer pressure, coworker support, culture, and role modelling. Codes were then iteratively grouped into sub‐themes, which were further refined into broader themes through constant comparison. Coding decision and theme development were discussed between reviewers to ensure clarity and consistency. Quotes were selected to support, exemplify, and give voice to the identified themes. They represent common sentiments found in the included qualitative data and provide real‐life examples, adding context and interest to the findings. Ethical approval was not needed as no new data was collected as part of this review.

## 3 RESULTS

**FIGURE 1:**
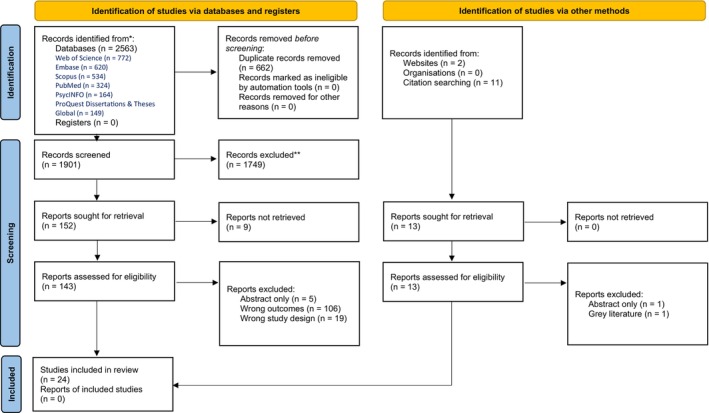
PRISMA 2020 flow diagram for new systematic reviews which included searches of databases, registers and other sources. ^*^Consider, if feasible to do so, reporting the number of records identified from each database or register searched (rather than the total number across all databases/registers). ^**^If automation tools were used, indicate how many records were excluded by a human and how many were excluded by automation tools. *Source*: Page MJ, et al. BMJ 2021;372:71. https://doi.org/10.1136/bmj.n71. This work is licensed under CC BY 4.0. To view a copy of this licence, visit https://creativecommons.org/licenses/by/4.0/.

The results of the literature search and screening process are shown in Figure 1. The search yielded 2563 results. After removal of duplicates, 1901 articles were title and abstract screened. One hundred and fifty‐two articles were sought for retrieval for full text review with nine articles unable to be retrieved. After full‐text assessment, 13 studies remained and were subjected to backwards and forwards citation searching. An additional 13 citations were identified via forwards and backwards citation searching and subject to the screening process, yielding another 11 studies that met the inclusion criteria. In total, 24 studies, dating from 2004^40^ to 2025,^41^, ^42^, ^43^ were identified to inform the review.

The key characteristics and findings of the 24 included studies are shown in Table 1. Twenty of the included papers were peer‐reviewed articles published in scholarly journals—seven quantitative,^40^, ^43^, ^44^, ^45^, ^46^, ^47^, ^48^ four mixed methods,^7^, ^16^, ^41^, ^42^ and nine qualitative studies.^14^, ^15^, ^49^, ^50^, ^51^, ^52^, ^53^, ^54^, ^55^ Four of the included studies were PhD dissertations: two quantitative,^30^, ^56^ one qualitative,^30^, ^57^ and one mixed methods.^58^ All but two studies^41^, ^42^ sampled full‐time career firefighters, who were predominantly white males with a median age between 39 and 51. Other study populations included wildland firefighters who were either temporary or permanent employees,^41^ participants who self‐reported current or past work in unspecified fire services,^42^ and career firefighters who were working as fire academy instructors and health and wellbeing staff.^54^ Two studies included both volunteer and career firefighters^50^, ^57^; another included both full‐time and part‐time career firefighters.^55^ Two studies also included the firefighters' partners.^30^, ^53^ Participants ranged in rank and length of service from recruit (less than 1 year's service)^7^ to fire chiefs with up to 39 years' service.^14^ Twenty‐one studies were undertaken in North America (one included data from Taiwanese firefighters)^43^; one was conducted in Canada,^48^ one in the United Kingdom^47^ and one in Australia.^7^

**TABLE 1:** Summary of included studies’ characteristics and findings, listed in descending publication order.

Author (year) country	Study aims	Study setting	Study design	Participant characteristics	Data collection & analysis	Findings
Lan et al.^43^ (2025) Taiwan and USA	To develop and validate a 24‐item organisational health culture measurement tool for firefighting workplaces	Career firefighters from the Tainan City Government Fire Bureau, Taiwan and recent graduates of Connecticut Fire Academy, USA.	Quantitative Cross‐sectional survey	Career firefighters (Taiwan) *N* = 435 Male = 397; female = 38 *M* age = 40.6 Recruit firefighters (USA) *N* = 28 Male = 25; female = 3 *M* age = 29.4	Statistical analysis of quantitative data from a 35‐item questionnaire using a 5‐point Likert scale, originally administered in traditional Chinese (the participants’ native language) and then translated to English in collaboration with local fire departments.	The 34‐item tool is a valid tool for assessing organisational health culture in fire services The tool assesses organisational health culture over six domains, including physical activity and nutrition policy, and includes the item “Healthy eating is discouraged in our firehouse”
Horton Dias et al.^55^ (2024) USA	To understand the occupational influences on firefighters’ dietary behaviour to inform nutrition interventions	Full time and part time career firefighters working 24 h shifts in South Carolina, USA	Qualitative Cross‐sectional focus groups and one semi‐structured interview	Full‐time and part‐time firefighters *N* = 34 Male = 35 *M* age = 34 years *M* LOS = 13.5 years	Thematic analysis of qualitative data guided by the Theoretical Domains Framework (TDF) and Consolidated Criteria for Reporting Qualitative Research (COREQ) guidelines	Social/professional role and identity and social influences were two of the predominant TDF domains found to influence firefighters’ (FFs) workplace dietary behaviours and thus emerged as targets for future interventions Specific social influences found to influence dietary behaviour included: conceptualisation of the work unit as a “brotherhood”, peer behaviour, role modelling, crew preferences, and social norms that were unique to each shift and station Eating together strengthened camaraderie Dinner was the most peer‐influenced meal because it was a group decision Fire service leadership played an important role in setting social norms and station culture
Holubetz et al.^41^ (2024) USA	To describe the current state, identify topics of importance, and suggest implementation strategies for tailored nutrition education for wildland firefighters.	Wildland firefighters representing all North American Forest Service regions	Mixed methods Cross‐sectional Focus group Survey	Wildland firefighters, temporary (n = 18) and permanent (n = 9) *N* = 27 Male = 18; female = 9 Age = 18‐54 years *M* LOS = 7.6 seasons	Thematic analysis of focus group transcripts Descriptive statistical analysis of quantitative survey data	Peers with an interest in nutrition and health were popular sources of nutrition information and role models Crewmember peer educators were identified as potential sources of nutrition guidance Conversing with colleagues about nutrition during meals/food preparation proposed as nutrition education strategy
McLanahan et al.^42^ (2024) USA	To characterise experiences with cancer and perceptions of the preventable cancer burden attributable to diet among firefighters.	North American firefighters	Mixed methods Cross‐sectional Survey	Participants who self‐reported current or past work in the fire service *N* = 471 *M* LOS = 22.6 years	Statistical analysis of quantitative survey data Descriptive statistics and Behaviour Change Technique Taxonomy evaluation of qualitative survey responses	Respondents expressed desire for social support for dietary change, that is, “I would need the entire crew to be on board with the diet change so our crew meals would follow a more sound diet” (p. 304) Respondents needed “information about others’ approval” (p. 305) for dietary change
Sire et al.^48^ (2024) Canada	To quantitatively investigate factors associated with firefighters’ occupational food behaviours	Career firefighters from 25 fire departments in Quebec, Canada	Quantitative Cross‐sectional Survey	Career firefighters *N =*252 Male = 241; female = 11 *M* age = 39 years *M* LOS = 16 years	Statistical analysis of quantitative data from a five‐part questionnaire using validated tools adapted for firefighters	Healthy eating support from colleagues was positively and significantly associated with participants’ healthy food score, but did not predict healthy food score after accounting for autonomous motivation Colleagues’ unsupportive actions related to healthy eating, fire chiefs’ healthy eating recommendations, number of colleagues and colleagues’ age were not associated with healthy food score Colleagues’ unsupportive healthy eating actions were positively and significantly associated with fast‐food score
Hershey et al.^46^ (2023) USA	To evaluate the effect of a Mediterranean nutrition intervention using a behavioural/environmental approach	52 fire stations of 2 Indiana Fire Departments	Quantitative Cluster randomised crossover trial	Career firefighters *N* = 485 (Control (*n* = 240); Intervention (*n* = 244)) Male = 458; female = 27 *M* age = 47 years	Statistical analysis of survey, anthropometric and blood pathology data	Adherence to modified Mediterranean diet score significantly increased (>10%) in intervention group at 6 and 12 months Intervention group increased vegetable, fish and legume consumption and improved nutritional quality of non‐alcohol beverages at home
Headrick^30^ (2021) USA	Test two models examining the interplay between emotional demands, unhealthy eating and substance use.	Midwest, USA	Quantitative Longitudinal multi‐wave survey	Career firefighters *N* = 228 Male = 212; female = 16 *M* age = 33.5 years *M* LOS 7.97 years Firefighters' partners *N* = 228 Male = 17; female = 211 *M* age = 32 years	Statistical analysis of 6 electronic questionnaires completed at different timepoints in shift schedule. Measures included perceived healthy eating climate, healthy eating norm, partner healthy eating support, and unhealthy eating.	Partner support for healthy eating and the perception of a healthy diet norm among coworkers buffered the relationship between negative affect at the end of shift and unhealthy eating off‐duty. FFs with high partner support for healthy eating, and whose coworkers ate more healthily, were less likely to eat unhealthy foods when negative affect was high than those with low partner/coworker support for healthy eating. Perceived healthy eating climate at work did not impact off‐duty unhealthy eating
Lessons and Bhakta^47^ (2021) UK	Design, implement and evaluate the feasibility and efficacy of a kitchen‐based cookery workshop intervention to ameliorate the fire station food environment.	Hornchurch Fire Station, London	Quantitative Prospective cluster‐controlled trial	Mess managers (firefighters responsible for food catering for their watches) *N* = 17 Male = 16; female = 1 *M* age = 45.2 years *M* LOS = 18.9 years	Statistical analysis of pre and post 9‐item food environment questionnaire and participant satisfaction rating	Individual FFs who cook for others and control the food environment can significantly change the food environment in their station, increasing health promoting behaviours
Garcia Rivas^56^ (2019) USA	To investigate individual, occupational, and environmental factors that play a role in the development of obesity in firefighters	Orange County Fire Authority, California	Quantitative Cross‐sectional Anthropometry Geographical data Survey	Career firefighters *N* = 365 Male = 356; female = 9 *M* age = 42.3 years (18–61)	Statistical analysis of anthropometrical measures, geographical data and survey on worksite characteristics, demographics & health behaviours including diet quality, eating habits and fast‐food consumption	Organisational support predicts nutrition self‐efficacy, but supervisor and coworker support do not. Social support buffered obesity outcomes by improving self‐efficacy
Sotos‐Prieto et al.^54^ (2019) USA	To evaluate the cultural barriers and challenges that might be faced upon implementing a diet or lifestyle modification program in a fire academy training setting	10 North American firefighter training academies; 1 national volunteer firefighter organisation	Qualitative Semi‐structured telephone interviews Online focus group	Fire academy staff members Interview sample *N* = 12 Male = 11; female = 1 *M* age = 49.2 years Focus group sample *N* = 5 Male = 5 *M* age = 50.8 years	Thematic analysis of interview and focus group transcripts	Rookie FFs influenced by peer pressure from senior FFs and have little power in deciding what to eat Fire training academy staff felt they should lead by example when it comes to healthy eating FFs responsible for cooking or serving food influenced others’ choices
Muegge et al.^53^ (2018) USA	To assess firefighter and firefighter partner perceptions of food, nutrition behaviour and culture	Indianapolis Fire Department, Indiana	Qualitative Semi‐structured focus groups	Career firefighters (8 privates, 4 lieutenants, 2 engineers and 1 captain) *N* = 15 *M* age = 46.6 years *M* LOS = 19.6 years	Thematic analysis of focus group transcripts	Motivators for dietary change include role modelling and co‐worker support Food choices at the firehouse depend on the designated cook FFs who pack a healthy meal to eat separately from their shift face scrutiny
Anderson et al.^49^ (2017) USA	Describe how firefighters perceive their cancer risk	15 Palm Beach County Fire Rescue stations, mix of urban and rural stations in South Florida	Qualitative Observation Focus groups	Observation sample: Career firefighters of all ranks *N* = >100 Focus group sample: Career firefighters (3 drivers, 4 non‐ranked firefighters, 6 captains and lieutenants, 4 specialty captains) *N* = 17 *M* age = 51 (29–58)	Ethnographic journalling Thematic analysis of focus group transcripts using grounded theory	Chefs/cooks influence the nutritional quality of the meal Some FFs attempt to persuade health‐conscious FFs to cook in order to eat healthier FFs face social sanctions if they want to change the food they eat on shift or bring their own food
Bonnell et al.^7^ (2017) Australia	Investigate the differences in dietary intake between night shift and day shift in metropolitan firefighters	Metropolitan fire stations in Melbourne	Mixed methods Cross‐sectional Focus groups Survey	Career firefighters *N* = 41 Male = 40; female = 1 *M* age = 46 years	Thematic analysis of focus group transcripts Statistical analysis of survey data	Observing colleagues purchase takeaways led to copycat behaviour FFs interested in cooking encouraged others to cook instead of buy food The presence of health and fitness advocates encouraged healthy choices
Kniffin et al.^16^ (2015) USA	Investigate organisational benefits that firms might obtain through various supports for coworkers to engage in commensality (i.e., eating together)	13 firehouses in a large city	Mixed Methods Cross‐sectional Semi‐structured group conversations and observation Survey	Qualitative sample: career firefighters of all ranks, including officers, on duty at 13 fire houses Quantitative sample: *N* = 244 Male = 237; female = 7	Ethnography Thematic analysis of enthographic notes and semi‐structured group conversations Statistical analysis of survey data	The importance of group cooking and eating within firehouses scored 8.07/9 Firehouses that did not do communal cook‐ups were regarded with embarrassment as people who “don't follow the tradition” (p. 296).
Wooding^57^ (2014) USA	Explore facilitators and barriers of healthy eating and physical activity in firefighters	West Virginia career and volunteer fire departments	Qualitative Cross‐sectional Focus groups Observation Photovoice	Career firefighters *N* = 27 Volunteer firefighters *N* = 31 Male = 54; female = 4 *M* age = 39 (19–66 years)	Consensual qualitative research analysis Ethnographic data analysis	Volunteer FFs cook more for themselves and eat more at home, rather than relying on others in the department to cook for them. Volunteers may not benefit from healthy eating behaviours of others in the department.
Dobson et al.^14^ (2013) USA	Investigate occupational and health behavioural determinants of obesity among firefighters	62 Orange County Fire Authority fire stations in 22 cities in Southern California	Qualitative Cross‐sectional Focus groups	Career firefighters (11 unranked firefighters/engineers, 4 captains, 5 battalion chiefs) *N* = 20 Male = 19; female = 1 LOS = 2–39 years	Thematic analysis of focus group transcripts using grounded theory	When eating out, FFs go along with the group; healthier options often unavailable FFs fear social sanctions for under‐catering/providing foods that are not ‘filling’ New FFs encouraged to overeat to prove themselves and win respect of older FFs
Frattaroli et al.^50^ (2013) USA	Understand cardiovascular disease risk among volunteer firefighters and use data to design a healthy lifestyle intervention	37 fire stations in Maryland, excluding Baltimore City	Qualitative Cross‐sectional Focus groups	Firefighters (75 volunteer only, 11 both paid and volunteer, 7 paid only, 3 other, 1 emergency medical services only, 1 unspecified) *N* = 98 Male = 83; female = 15 *M* age = 41 years *M* LOS = 19 years	Coding of focus group transcripts	Peer health advocates and weight loss competitions seen as viable interventions Food influenced by FFs home, family, fire station and colleagues
Mabry et al.^52^ (2013) USA	Explore influences associated with durability and diffusion of benefits of the PHLAME^40^ wellness program.	48 fire stations	Qualitative Assessment of interviews from PHLAME study^40^	Firefighters *N* = 68 PHLAME participants (*n* = 51) New employees (*n* = 17) *M* age = 46 years	Thematic content analysis	Individual dietary change influenced group attitudes and behaviours; group change influenced individual behaviours Group norms changed by the intervention, and “outliers” (non‐conforming FFs) teased into acceptance of new norms (p. 698) Newly hired FFs brought generational change through increased health knowledge and motivation for healthy eating
Jahnke et al.^51^ (2012) USA	Obtain the perspectives of fire service personnel in regard to understanding firefighters’ increased risk for disease and injury	28 municipal and federal career fire departments across North America	Qualitative Cross‐sectional Interviews Focus groups	Career firefighters, officers, fire chiefs and other fire service staff *N* = 332 Male = 309; female = 23 *M* age = 41.7 years *M* LOS = 15.6 years	Thematic analysis of focus group and interview transcripts	Social norms around eating a barrier to healthy lifestyles FFs expected to conform to meal‐time traditions or risk being ostracised Food choices were influenced by traditions of rich and unhealthy foods in large portion sizes
Ranby^45^ (2011) USA	Examine the mechanisms by which PHLAME^40^ improved healthy eating and exercise behaviour among firefighters	48 fire stations	Quantitative Randomised controlled trial	Career firefighters N = 397 Male = 369; female = 28 *M* age = 41 years	Pre and post (1 year) dietary intake, physiological measures, anthropometry measures, perceived coworker support Statistical analysis	Team participants had increased fruit and vegetable consumption Improved dietary environment among coworkers
Kipp^58^ (2008) USA	To understand the perceived influences on healthy behaviours among a group of career firefighters	Firefighters in Corvallis, Oregon	Mixed Methods Cross‐sectional Focus groups Anthropometry Survey	Career firefighters *N* = 58 Male = 56; female = 2^a^ *M* age = 39 years (25–55)	Grounded theory Thematic analysis of focus group transcripts Descriptive statistical analysis of anthropometry and survey data	Peer influences caused FFs to eat more and eat less healthy food. Peer influenced encouraged FFs to stay in shape so as not to ‘let other FFs down’ (p. 30) Peer influences had both positive and negative impacts on FFs’ health behaviours
Elliot et al.^44^ (2007) USA	To assess and compare the efficacy of two worksite health promotion interventions	Fire stations in close proximity to Oregon Health & Science University	Quantitative 3 arm randomised controlled prospective trial	Career firefighters *N* = 599 (Control *n* = 163 vs. team intervention *n* = 234 vs. individual intervention *n* = 202) Male = 579; female = 20 *M* age = 41 (20–60)	Pre and post (1 year) dietary intake, physiological measures, anthropometry measures, perceived coworker support Statistical analysis	Both team and individual interventions positively impacted nutrition behaviours (significant increase in fruit and vegetable servings/day) and were associated with less weight gain and enhanced general well‐being compared to control The team curriculum increased dietary social support Explicit team‐based nutrition activities were necessary to recruit positive social influences
Deutsch^15^ (2005) USA	Investigate the dynamics of non‐food‐professional men who are the primary cooks for the men of an urban firehouse	An urban firehouse in North America	Qualitative Observation Semi‐structured interviews	Career firefighters from 1 engine company and 1 ladder company *N* =14 Male = 14 Age = 25–54	Ethnographic notes transcribed using the method of Patai (1988) and thematically analysed	Cooking is a family act for the firehouse “family” and outsiders are not allowed to contribute. Meals chosen based on what is perceived to appeal to the majority FFs may “count themselves out” of a group meal but this is akin to counting themselves out of the group altogether and is rarely done (p. 109)
Elliot et al.^40^ (2004) USA	To assess the efficacy of two worksite health promotion interventions	3 fire stations in North America	Quantitative 3 arm randomised controlled trial	Career firefighters *N* = 599 (Control *n* = 163 vs. team intervention *n* = 234 vs. individual intervention *n* = 202) Male = 579; female = 20 *M* age = 41 (20–60)	Pre and post (1 year) dietary intake, physiological measures, anthropometry measures, perceived coworker support Statistical analysis	Shift eating habits improved significantly in the team intervention group but not the individual intervention group or control The team‐based curriculum increased shift cohesion Only the team group perceived a significant increase in colleagues’ healthy dietary habits

Abbreviations: FFs, firefighters; LOS, length of service; *M*, mean; *N*, sample size, TDF, Theoretical Domains Framework.

^a^ Women's responses were removed from analysis as they were administrative staff, not operational firefighters.

The included studies suggested that the social influence of colleagues is a key factor in firefighters' dietary behaviour. Specific social influences found to influence dietary behaviour included the behaviour, knowledge‐sharing and preferences of peers; teasing and social exclusion; role modelling; social/professional identity, and social norms. Behaviours that appeared subject to change because of social influence included firefighters' food choices, portion sizes, reasons for eating, expression of food preferences and communal meal suggestions. It was found that social influence could be either negative (toward unhealthier eating patterns) or positive (toward healthier eating patterns). Social influence could be exerted by a group of firefighters onto an individual, by an individual on another individual, or by an individual onto a group. The social influences identified across the included studies were grouped into two emerging themes: (i) culture and tradition and (ii) people and positions of influence.

Culture refers to a way of thinking, behaving, or working that exists in a place or organisation.^59^ A fire service's ‘organisational health culture’ impacts on its employees' health behaviours, including dietary behaviour.^43^ Culture is largely dictated by social norms, which are the unwritten rules that define appropriate and expected behaviour within a specific group.^60^ Across the studies included in this review, the social norms that influenced firefighters' occupational dietary behaviours were characterised as “rich in structure and tradition”^53(p3^ ^)^; “uniquely resistant to change”,^51(p8^ ^)^ and often considered a barrier to positive dietary change.^49^ Fire service leadership played a key role in setting social norms, which could be unique to each shift and station.^55^ Examples of social norms included:

1. Participation in communal or ‘family‐style’ meals, which involved eating larger‐than‐usual portions of foods lower in nutritional quality^7^, ^14^, ^58^;
2. Conforming with ‘group consensus’ on meal choice regardless of personal preference^7^, ^14^, ^51^;
3. Buying desserts high in fat and sugar to celebrate the commencement of new firefighters and ‘firsts’ (i.e., a firefighter's first fire), or to punish mistakes, accidents, or poor performance by forcing the offending firefighter to buy desserts for their crew.^14^ These desserts were traditionally consumed as a group in the evenings, forming part of the social support environment of the fire station.^14^

Hershey et al.'s^46^ randomised crossover trial showed that a healthy eating intervention designed to leverage the social support of family and colleagues increased firefighters' adherence to the Mediterranean diet and improved their dietary quality. The earlier randomised controlled trial by Elliot et al.,^40^ the Promoting Healthy Lifestyles: Alternative Models' Effects firefighters' study (PHLAME), also successfully facilitated improved firefighters' dietary quality, specifically by eliciting social norm change through a group‐focused intervention. The PHLAME study found that there was a bidirectional effect of social influence between individuals and groups that facilitated and reinforced positive dietary behaviour change, whereby individuals catalysed group change in some cases, and in others, group change brought about individual‐level change.^52^

Social exclusion from the tight‐knit firefighter ‘family’ was a feared consequence of not conforming to the social norms governing dietary behaviours in firefighters' occupational contexts.^7^, ^14^, ^15^, ^49^, ^51^ The threat of social exclusion motivated firefighters to participate in communal meals at the fire station or go along with the group consensus regarding takeaway food choices, even if those choices were not consistent with their personal preferences or values:

“If you're at a station where they do cook together there's a pretty big culture around it so you don't want to be a splitter and not eat with them so yeah, you tend to have bigger meals and maybe not as healthy because you don't want to not eat with them.”^7(p7^ ^)^

Communal mealtimes were regarded as a ‘sacred’ time for sharing stories and bonding,^49^, ^55^ and of such importance that firefighting crews who “don't follow the tradition” of communal cooking were regarded with suspicion and embarrassment.^16^ Likewise, individuals who brought a meal from home instead of participating in the communal ‘cook‐up’ faced scrutiny “like sharks on a minnow”^53(p3^ ^)^ and could expect to experience social sanctions such as being ostracised^51^ or “counted out”.^15(p109^ ^)^ All such consequences were conscientiously avoided ‐ even when disguised as humour or good‐natured teasing:

“Firefighters… exhibited a tendency to avoid conflict and even disagreement among themselves. Jollying outliers into the fold, they amicably but relentlessly joked and teased each other into acceptance of group norms.”^52(p698^ ^)^

Many firefighters described consciously altering their dietary behaviours to avoid being laughed at,^7^ given “a little bit of lip”,^58(p31^ ^)^ having to “hear about it for a long time”,^14(p780^ ^)^ or being “crucified”.^58(p31^ ^)^ In one study, firefighters characterised teasing as a form of motivation to maintain health behaviours (i.e., to avoid the embarrassment of being teased about an increase in uniform size).^58^ More often, however, the motivation to avoid social sanctions led firefighters to make negative dietary changes, such as eating or preparing more food than they usually would, or wanted to; changing their food choices to be more in line with colleagues', despite knowledge that those choices were less healthy; moderating their meal suggestions, and concealing their true food preferences^7^, ^14^, ^58^:

“You don't want to be the odd one out, so if everyone wants to get salad rolls you're hardly gonna (sic) say well no I want Maccas (McDonalds) or I want pizza, but then if like 2 or 3 guys go ‘ohh let's get pizza tonight boys’ you don't want to be the guy who goes nah I'd rather have a salad roll…”^7(p8^ ^)^

Inversely, dietary behaviour could also be used to accept new members to the group and make them feel part of the ‘family’, functioning as a “proving grounds for the new recruits”^15(p94^ ^)^:

“When I got hired and I went to this one station, there were 15 guys on and… they were all huge… you are kind of nervous about how much you are supposed to eat and when, and all that stuff. So one guy… said ‘hey, you really want to impress all these guys? Eat as much food as you can, stuff it down as fast as you can, they will give you another plate and you will really impress these guys.’ … I started doing that and they said [he's alright] from day one. And I remember getting a call afterwards and I had to spit out all my food because it was almost twofold more than I [usually] eat.”^14(p780^ ^)^

This example illustrates how social norms are communicated from generation to generation, creating traditions that become part of firefighters' workplace culture. The studies included in the scoping review indicated that these traditions, passed on by senior firefighters, often negatively influence the dietary behaviour of recruit (‘rookie’) firefighters.^14^, ^54^ Sotos‐Prieto et al.^54^ interviewed fire academy trainers about the transition of new firefighters from the academy to the fire station and found that recruits' dietary behaviours declined once they left the academy. This was due to the absence of the positive influence of academy trainers who “try to set the example” by discouraging poor‐quality food choices.^54^ Instead, senior firefighters told recruits “don't bother cooking anything healthy, we're a meat and potatoes shift” or role modelled “going off‐site (to) eat fast food pretty commonly”.^54(p777^ ^)^ Dobson et al.^14^ noted that senior colleagues taught recruits to cook “big and bland” meals that were cheap and filling. These practices served to teach and reinforce dietary behaviours that helped recruit firefighters become accepted into their new ‘family’, but simultaneously caused them to feel powerless over their occupational food choices:

“When you get into a station as a new rookie and you're told to keep your mouth shut and do as you're told and you know sit at the table and eat with your crew and you don't dare tell the crew, ‘I'm not going to eat that because it's not healthy”.^14(p780^ ^)^

Conversely, new firefighters exerted social influence in some fire stations, influencing their colleagues to make healthier choices: In “The younger ones have knowledge, and they want to be healthier. They are eating healthier, and they're trying to motivate the rest”.^52(p699^ ^)^ The perception of health knowledge was one of the individual characteristics or roles that facilitated individual firefighters' exertion of social influence over other firefighters' dietary behaviour, according to the reviewed studies.^7^, ^14^, ^52^, ^58^ Peers were a popular source of nutrition information,^41^ and peer‐led nutrition interventions were called for by firefighters^50^, ^55^ and found to be effective when implemented.^40^, ^46^ Firefighters who were perceived to have specific expertise or interest in health, fitness and/or cooking^7^, ^14^, ^41^ exerted positive influence on their colleagues' dietary behaviour. This influence could be exerted passively or actively, with an example of passive exertion being:

“We actually have a very passionate personal trainer at our station… to the point where when he's on shift people tend to eat healthier just because they don't want to have to have that confrontation where they have to explain what they're eating to him”.^7(p9^ ^)^

Other firefighters actively influenced dietary behaviour, for example by “forcing more of the kale, and… the double vegetable and lack of potato”.^49(p648^ ^)^ Anderson et al.^49^ found that some firefighters tried to persuade these active influencers ‐ whom they termed “health guys” ‐ to take responsibility for meal preparation, as a healthy communal meal was seen as preferable to “skipping unhealthy communal meals and eating alone”.^49(p648^ ^)^ Individuals involved in food procurement and preparation processes in the fire station exerted influence over their colleagues' dietary behaviours.^15^, ^41^, ^53^, ^54^ A prospective cluster‐controlled trial showed that individuals who were permanently responsible for overseeing the food environment in the fire station (“mess managers”) could influence the dietary behaviour of groups of firefighters by changing the types of foods that were available at the station and the recipes used in cooking.^47^

Firefighters who were temporarily tasked with preparing food for their crew also exerted this influence,^15^, ^53^, ^54^ suggesting the influence is attached to the role of ‘cook’, rather than to specific individuals. Qualitative studies provided insight into the nature of the cook's influence and highlighted that it could be negative or positive.^53^, ^54^, ^57^ Wooding^57^ found that volunteer firefighters may not benefit from other firefighters' healthy eating behaviour because they were more likely to cook for themselves and eat at home instead of relying on other firefighters to cook for them, as career firefighters did. However, career firefighters who depended on others to cook for them were not guaranteed to receive a healthy meal.^7^, ^49^, ^53^, ^58^

“For 24 hours, it's like I may be at the mercy of what Bob wants to cook unless I want to bring my own meal.”^53(p3^ ^)^

On‐duty firefighters buying pre‐prepared meals outside the fire station could also influence others' dietary behaviour^48^:

“If we go out and someone else has to buy lunch and I like what they're getting, I'll forget what I took into work and I'll say ‘I'll have some of that!’ That's how I have more takeaway”.^7(p8^ ^)^

## 4 DISCUSSION

This scoping review analysed the findings of 20 peer‐reviewed papers and four PhD dissertations to explore the literature on social influences on firefighters' occupational dietary behaviour. Across the included studies, two key themes were identified: culture and social norms, and people and positions of influence. Social norms are the foundation of culture^61^ and firefighters have a distinct workplace culture.^14^, ^51^, ^53^, ^54^ The findings of this scoping review suggest that firefighting colleagues influence each other to conform with the social norms that govern dietary behaviours in their specific workplace culture. Senior firefighters teach recruit firefighters the social norms of the firefighting workplace through a process termed ‘acculturation’.^15^ Noncompliance with these norms may result in social consequences including social exclusion from groups of colleagues and teasing. Accordingly, firefighters model their dietary behaviours on their colleagues' to avoid such sanctions, a pattern consistent with the proposition that people conform to eating social norms in order to belong to a group and to avoid social penalties.^21^, ^26^ The findings of this review support previous literature recognising the social influence of colleagues as a key factor in dietary behaviours among night shift workers^24^, ^25^, ^26^, ^29^, ^31^, ^62^ and emergency first responders,^27^, ^28^, ^32^ noting that these influences can have both positive and negative impacts on dietary quality. This review extends previous research by describing *how* dietary social norms within firefighting workplaces are communicated among colleagues, and by providing detailed insights into the roles and characteristics of firefighters who exert or are vulnerable to social influence.

Cooks, health and fitness advocates, and in some cases recruit firefighters exerted social influence over the dietary behaviours of their colleagues, either toward healthier or less healthy eating patterns, depending on the influencer. Recruit firefighters were identified as a positive influence due to their health knowledge.^52^ However, they were also particularly vulnerable to changing their dietary behaviours toward less healthy eating patterns to conform with social norms. This aligns with existing literature which describes norm‐following as more common where there is norm uncertainty^21^ and among those on the periphery of a social group^22^ such as a new employee. It also aligns with research in other close‐knit social groups, such as adolescents. Young people who feel less secure in their social group and have a higher desire for peer approval consume less vegetables,^63^ as healthy eating conflicts with the self‐image teenagers wish to portray in order to affiliate with their desired peer group.^64^

Firefighters with a personal interest in health, fitness, or cooking often positively influenced their colleagues' dietary behaviour. Firefighters who controlled the food environment in the fire station were also influential, either negatively or positively. Compelling dietary behaviour research suggests that the availability of healthy and less healthy foods, as well as social support and role modelling, are important in determining dietary behaviours.^65^ Role modelling of health behaviours among colleagues has been shown to promote behaviour change.^66^ Accordingly, future research could target these people and positions of influence as conduits to improving dietary behaviours in firefighting workplaces. Mess managers, cooks, health and fitness enthusiasts and recruits could be used as ‘peer health advocates’, an approach that has been found to be effective in workplace health interventions among firefighters^67^ and law enforcement officers.^68^ This strategy has been suggested by firefighters as a preferred workplace health promotion intervention.^41^, ^50^

The recruit training process presents an opportunity to increase new firefighters' awareness of the dietary social norms likely to be encountered in fire stations and the potential health impacts of those norms. Recruits could be educated on appropriate responses to the actions and attitudes of senior colleagues; taught validated techniques for motivating behaviour change; and given practical skills in cooking healthy meals that meet firefighters' nutritional needs. Concurrent efforts could be directed at educating senior firefighters, who are considered cultural leaders in the workplace,^69^ to strengthen healthy leadership in firefighting workplaces and break down cultural barriers to change.

The findings of this review, together with existing literature on social influences on dietary behaviour in other occupations, highlight the dual nature of social influence as being capable of reinforcing unhealthy eating patterns as well as fostering positive change. What distinguishes firefighters from other shift‐working groups is the cultural and traditional importance of communal eating practices and the centrality of food to occupational identity. These factors make social influence both a greater barrier and a greater opportunity for dietary interventions in firefighting workplaces.

The distinct cultural and practical (i.e., the availability of time and facilities for cooking on‐shift) differences between firefighting and other professions may affect the generalisability of these findings. The quality of the included studies was not evaluated, consistent with the methodological scope of scoping reviews, which aim to map rather than appraise evidence. Additional limitations include the homogeneity of the samples, although they were generally representative of the demographics of the profession. Most studies were conducted in the United States, and representation of volunteer and on‐call firefighters was low. Consequently, the findings may not capture the existing breadth and variety of firefighting workplaces.

In conclusion, this scoping review highlights the central role of social influence in shaping firefighters' occupational dietary behaviours, particularly through workplace culture, social norms, and key individuals in positions of influence. These dynamics can act as both barriers and facilitators to healthy eating, underscoring the need for contextually tailored interventions. The unique cultural importance of communal eating in firefighting workplaces presents a powerful opportunity to leverage social influence for positive dietary change. Future research and workplace health initiatives should consider these social mechanisms to support sustainable improvements in firefighters' nutrition and overall health.

## AUTHOR CONTRIBUTIONS

HR was the primary designer of the work; AB, RO, and KMS contributed to the project conception. HR conducted the database searches. ML and HR conducted the data collection and analysis, including database searching, screening, and data charting. ML supplied the data extraction table. HR completed the data interpretation and drafted the work with input and supervision from KMS. HR, ML, AB, LVH, and KMS revised the work for intellectual content, grammar, phrasing, and formatting. AB, KMS, LVH, and RO provided final approval of the publishable version. All authors agree to be accountable for the accuracy and integrity of the work.

## FUNDING INFORMATION

The authors report that there was no funding source for the work that resulted in the article or the preparation of the article.

## CONFLICT OF INTEREST STATEMENT

HR was an on‐call firefighter for a metropolitan fire service at the time of writing.

## ETHICS STATEMENT

This work was carried out at Bond University, Gold Coast, Australia. Ethical approval is not required for this scoping review as it is secondary research that does not involve human participation or the collection of personal, sensitive or confidential information.

## Supporting information


**TABLE S1:** Search strategy applied to electronic databases.

## Data Availability

Data sharing not applicable to this article as no datasets were generated or analysed during the current study.
