# Supplementary material for: Social influences on the occupational dietary behaviour of firefighters: A scoping review
Source: Nutr Diet. 2026 Apr 20;83(2):220–40. doi: 10.1111/1747-0080.70076 (PMC13096670; doi:10.1111/1747-0080.70076)
Supplement: Supplementary file 1 — TABLE S1: Search strategy applied to electronic databases. [file NDI-83-220-s001.docx]

**Table S1.** Search strategy.

| **Database** | **Search lines** |
| --- | --- |
| PubMed | ("firefighter*"[tiab] OR "fire fighter*"[tiab] OR "fire and rescue"[tiab] OR “fire service”[tiab] OR “fire brigade”[tiab] OR “fire rescue”[tiab] OR "firefighters"[MeSH]) AND (“food”[tiab] OR “eating”[tiab] OR “nutrition”[tiab] OR “diet*”[tiab] OR “nutrient*”[tiab] OR “food choice*”[tiab] OR “Cardiovascular”[tiab] OR “Obesity”[tiab] OR “Weight”[tiab] OR “food preferences”[MeSH] OR “body weight”[MeSH] OR “body mass index”[MeSH] OR “Eating”[Mesh] OR “Nutrients”[Mesh] OR "Diet, Food, and Nutrition"[Mesh] OR “Diet”[Mesh] OR “Feeding Behavior/psychology"[Mesh] OR "Diet, Healthy"[Mesh] OR "Eating/psychology"[Mesh]) |
| PsycINFO (Ovid) | (exp Fire Fighters/ or firefighter*.ti,ab. or "fire fighter*".ti,ab. or "fire and rescue".ti,ab. or "fire service".ti,ab. or "fire brigade".ti,ab. or "fire rescue".ti,ab.) AND (exp Food Intake/ or exp Food/ or exp Eating Behavior/ or exp Body Weight/ or exp Obesity/ or exp Body Mass Index/ or food.ti,ab. or eating.ti,ab. or nutrition.ti,ab. or diet*.ti,ab. or nutrient*.ti,ab. or 'food choice*'.ti,ab. or Cardiovascular.ti,ab. or Obesity.ti,ab. or Weight.ti,ab. or exp food preference/ or exp Eating/) |
| Web of Science | Select ‘Topic’   (firefighter* OR "fire fighter*" OR "fire and rescue" OR "fire service" OR "fire brigade" OR "fire rescue") AND (food OR eating OR nutrition OR diet* OR nutrient* OR "food choice*” OR Cardiovascular OR Obesity OR Weight OR "food preferences" OR "body weight" OR "body mass index" OR "eating behaviour”) |
| Scopus | (TITLE-ABS(firefighter*) OR TITLE-ABS("fire fighter*") OR TITLE-ABS("fire and rescue") OR TITLE-ABS("fire service") OR TITLE-ABS("fire brigade") OR TITLE-ABS("fire rescue") OR INDEXTERMS(firefighters)) AND (TITLE-ABS(food) OR TITLE-ABS(eating) OR TITLE-ABS(nutrition) OR TITLE-ABS(diet*) OR TITLE-ABS(nutrient*) OR TITLE-ABS("food choice* “Cardiovascular”") OR TITLE-ABS(Obesity) OR TITLE-ABS(Weight) OR INDEXTERMS("food preferences") OR INDEXTERMS("body weight") OR INDEXTERMS("body mass index") OR INDEXTERMS(Eating) OR INDEXTERMS(Nutrients) OR INDEXTERMS("Diet, Food, and Nutrition") OR INDEXTERMS(Diet) OR INDEXTERMS("Feeding Behavior/psychology") OR INDEXTERMS("Diet, Healthy") OR INDEXTERMS(Eating/psychology)) |
| ProQuest Dissertations and Theses | (SUBJECT(“Firefighter" OR "fire and rescue service") OR ABSTRACT,TITLE(fire?fighter*) OR ABSTRACT,TITLE("fire and rescue") OR ABSTRACT,TITLE("fire service") OR ABSTRACT,TITLE("fire brigade") OR ABSTRACT,TITLE("fire rescue")) AND (ABSTRACT,TITLE(food OR diet* OR nutrition OR eating OR weight OR obesity OR cardiovascular) OR SUBJECT("Diet" OR "Diet - nutritional intake" OR "diet" OR "Eating" OR "Eating behavior and attitudes" OR "Eating decisions" OR "eating behavior" OR "eating" OR "eating behaviors" OR "Eating behavior regulation" OR "Eating behaviors" OR "Eating behaviours" OR "Eating choices" OR "eating culture")) |
| Embase (Elsevier) | (firefighter*:ti,ab OR 'fire fighter*':ti,ab OR 'fire and rescue':ti,ab OR 'fire service':ti,ab OR 'fire brigade':ti,ab OR 'fire rescue':ti,ab OR ‘fire fighter’/exp) AND ('food'/exp OR 'food preference'/exp OR 'body mass'/exp OR 'eating'/exp OR 'nutrition'/exp OR 'diet'/exp OR 'feeding behavior'/exp OR 'healthy diet'/exp OR 'cardiovascular disease'/exp OR 'food':ti,ab,de OR 'eating':ti,ab,de OR 'nutrition':ti,ab,de OR 'obesity':ti,ab,de OR 'weight':ti,ab,de) NOT [medline]/lim |
